# Feasibility and optimization of ^19^F MRI on a clinical 3T with a large field-of-view torso coil

**DOI:** 10.1088/1361-6560/ad4d50

**Published:** 2024-06-04

**Authors:** Lawrence M Lechuga, Monica M Cho, David M Vail, Christian M Capitini, Sean B Fain, Paul Begovatz

**Affiliations:** 1 Department of Medical Physics, University of Wisconsin School of Medicine and Public Health, Madison, WI, United States of America; 2 Department of Pediatrics, University of Wisconsin School of Medicine and Public Health, Madison, WI, United States of America; 3 Department of Medical Sciences, University of Wisconsin School of Veterinary Medicine, Madison, WI, United States of America; 4 Carbone Cancer Center, University of Wisconsin, Madison, WI, United States of America; 5 Department of Biomedical Engineering, University of Wisconsin School of Engineering, Madison, WI, United States of America; 6 Department of Radiology, University of Iowa, Iowa City, IA, United States of America

**Keywords:** fluorine-19, simulation, canine model, MRI

## Abstract

*Objective.* The objective of this work is to: (1) demonstrate fluorine-19 (^19^F) MRI on a 3T clinical system with a large field of view (FOV) multi-channel torso coil (2) demonstrate an example parameter selection optimization for a ^19^F agent to maximize the signal-to-noise ratio (SNR)-efficiency for spoiled gradient echo (SPGR), balanced steady-state free precession (bSSFP), and phase-cycled bSSFP (bSSFP-C), and (3) validate detection feasibility in *ex vivo* tissues. *Approach.* Measurements were conducted on a 3.0T Discovery MR750w MRI (GE Healthcare, USA) with an 8-channel ^1^H/^19^F torso coil (MRI Tools, Germany). Numerical simulations were conducted for perfluoropolyether to determine the theoretical parameters to maximize SNR-efficiency for the sequences. Theoretical parameters were experimentally verified, and the sensitivity of the sequences was compared with a 10 min acquisition time with a 3.125 × 3.125 × 3 mm^3^ in-plane resolution. Feasibility of a bSSFP-C was also demonstrated in phantom and *ex vivo* tissues. *Main Results*. Flip angles (FAs) of 12 and 64° maximized the signal for SPGR and bSSFP, and validation of optimal FA and receiver bandwidth showed close agreement with numerical simulations. Sensitivities of 2.47, 5.81, and 4.44 ${\mathrm{m}}{{\mathrm{s}}^{ - 0.5}}\,\,\,{\mathrm{m}}{{\mathrm{M}}^{ - 1}}{\text{ }}$ and empirical detection limits of 20.3, 1.5, and 6.2 mM were achieved for SPGR, bSSFP, and bSSFP-C, respectively. bSSFP and bSSFP-C achieved 1.8-fold greater sensitivity over SPGR (*p* < 0.01). *Significance.* bSSFP-C was able to improve sensitivity relative to simple SPGR and reduce both bSSFP banding effects and imaging time. The sequence was used to demonstrate the feasibility of ^19^F MRI at clinical FOVs and field strengths within *ex-vivo* tissues.

## 1. Introduction

Adoptive cell transfer (ACT) therapies for cancer have the potential to treat high burdens of disease and lead to sustained remissions and/or cures (Rosenberg 2001, Rosenberg *et al* 2008). While there are increasing numbers of clinical trials around the world testing ACT therapies, there remains high variability in clinical outcomes (Turtle *et al* 2016) and more limited success in solid tumors (Newick *et al* 2017). This is in part due to the inability to track and assess the biodistribution of the adoptively transferred cell population after treatment (Melero *et al* 2014, Sta Maria *et al* 2014). The United States Food and Drug Administration has published guidelines (Anon 2013) highlighting the need to incorporate cellular imaging to assess cell survival and biodistribution at all stages of therapeutic product development. Currently, there is a lack of immunotherapeutic-specific biomarkers that can assess early-stage treatment efficacy (Seymour *et al* 2017). Development of a non-invasive molecular imaging platform to track and quantify these cells *in vivo* throughout treatment can provide useful information to improve patient outcomes (Varani *et al* 2019).

Fluorine-19 (^19^F) MRI is one imaging modality that can be utilized to track and monitor *ex-situ* or *in-situ* labeled lymphocytes for days to weeks at a time. Labeling of these cells is accomplished by way of injection or *ex vivo* incubation with biologically inert perfluorocarbon (PFC) nanoemulsions (Janjic and Ahrens 2009). Given the low endogenous fluorine concentration in host tissue, resultant images will provide specific, positive contrast images, not corrupted by background signal. In the preclinical setting, this imaging modality has successfully tracked and labeled various immune cells such as macrophages (Temme *et al* 2012, Makela and Foster 2018), unmodified T-cells and chimeric antigen receptor-T cells (Srinivas *et al* 2007, 2009, Chapelin *et al* 2017, Hingorani *et al* 2020), natural killer (NK) cells (Bouchlaka *et al* 2016, Somanchi *et al* 2016, Lechuga *et al* 2021), and dendritic cells (DCs) (Waiczies *et al* 2011). There are considerably fewer cell tracking studies performed in a clinical setting, given several hurdles to clinical translation such as a lack of dedicated hardware (coils and broadband capabilities), lower achievable *in vivo* ^19^F concentration, and dedicated ^19^F sequences. However, one particular study (Ahrens *et al* 2014) demonstrated clinical applicability by tracking labeled adoptively transferred DCs in 5 patients over 24 h post-injection.

Although ^19^F MRI has intrinsically high specificity, the fluorine concentration that can be reasonably achieved *in vivo* leads to low sensitivity, which can lead to high signal averaging and excessive acquisition times. Efforts to improve sensitivity have included the use of high ^19^F molarity agents, such as perfluoropolyether (PFPE) (Srinivas *et al* 2007), compressed sensing (Zhong *et al* 2013) to reduce time constraints, wavelet-based denoising (Darçot *et al* 2019), and pulse sequence development. Among the clinically suitable ^19^F agents, PFPE is particularly attractive because of its high fluorine content and simple resonance spectrum. To maximize the signal-to-noise ratio (SNR) per square root of time, or SNR-efficiency (SNR_eff_), pulse sequences and their respective parameters need to be chosen carefully. For example, recent work from Colotti *et al* characterized the relaxation times of three common PFCs under various conditions and provided a theoretical parameter optimization of multiple pulse sequences at 3T using a pre-clinical small animal 35 mm birdcage coil (2017).

The feasibility and sequence optimization for ^19^F MRI using cellular tracking probes on clinical MRI systems is under-studied, especially for body imaging applications using a multi-channel array coil. Candidate fast, SNR-efficient sequences include fast spin-echo (FSE), variants of gradient recalled echoes, ultrashort echo time (Bönner *et al* 2015), and balanced steady-state free precession (bSSFP) (Rothe *et al* 2019). In bSSFP imaging (Bernstein *et al* 2005), the balanced gradients across a TR create a large steady-state magnetization that is particularly appealing for ^19^F MRI. As theoretically demonstrated in previous work (Flogel and Ahrens 2017), bSSFP produced the greatest SNR-efficiency for acquisitions under relaxation times in the ranges of the most common ^19^F probes, like PFPE and perfluoro-15-crown-5-ether (PFCE). While bSSFP’s signal efficiency and utility has been demonstrated for small animal MRI systems and birdcage coil designs in previous works (Goette *et al* 2015a, Colotti *et al* 2017), the well-known sensitivity to off-resonance artifacts of bSSFP (Hargreaves *et al* 2001), can become problematic for longer TRs and the reduced field homogeneity across the larger field of view (FOV) of clinical systems and applications. One strategy to reduce the off-resonance banding in bSSFP is known as phase cycling (Hargreaves 2012, Dubois *et al* 2022), where 2 or more acquisitions at different RF phase combinations are used to shift the location of signal nulls. Upon completion, the two acquisitions are combined in either a maximum intensity projection (MIP) or a sum-of-squares reconstruction (Chavhan *et al* 2008).

Human-sized single-channel birdcage coils for brain applications allow for simpler direct quantification, but they are not suitable for typical body imaging applications due to limited bore size and SNR. Surface coils offer increased sensitivity, with the trade-offs of limited fields of view and penetration depth that may preclude their use in some cell tracking studies.

**Figure 1.**
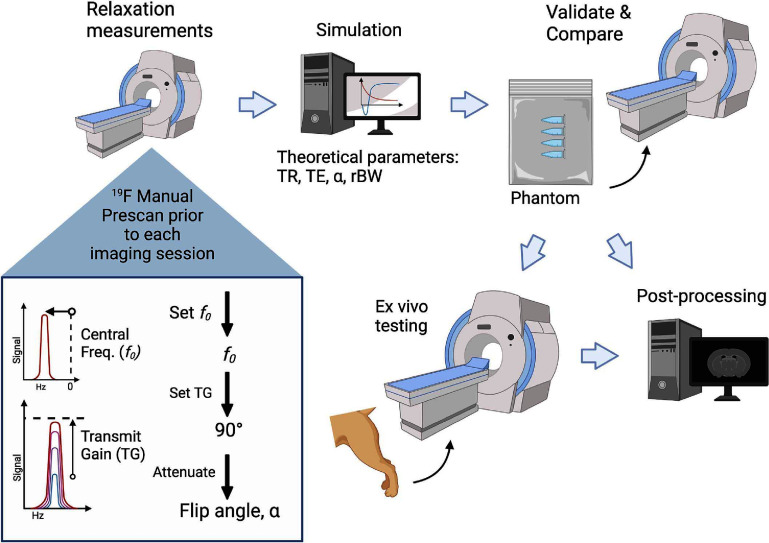
Graphical abstract demonstrating the experimental workflow. Created with permission from BioRender.com.

Demonstrated graphically in figure 1, the purpose of this work is to: (1) present a common workflow for optimizing the performance of PFC-compounds with respect to image SNR using a multi-channel torso array and apply this methodology to the performance of ^19^F MRI on a 3T clinical MRI platform using minimally modified product sequences, (2) verify the optimized parameter selection and compare the SNR-efficiency for SPGR, bSSFP, and phase-cycled bSSFP (bSSFP-C) using PFPE, and (3) validate detection feasibility in *ex vivo* tissues.

## 2. Methods

### 2.1. MRI setup and measurements

**Figure 2.**
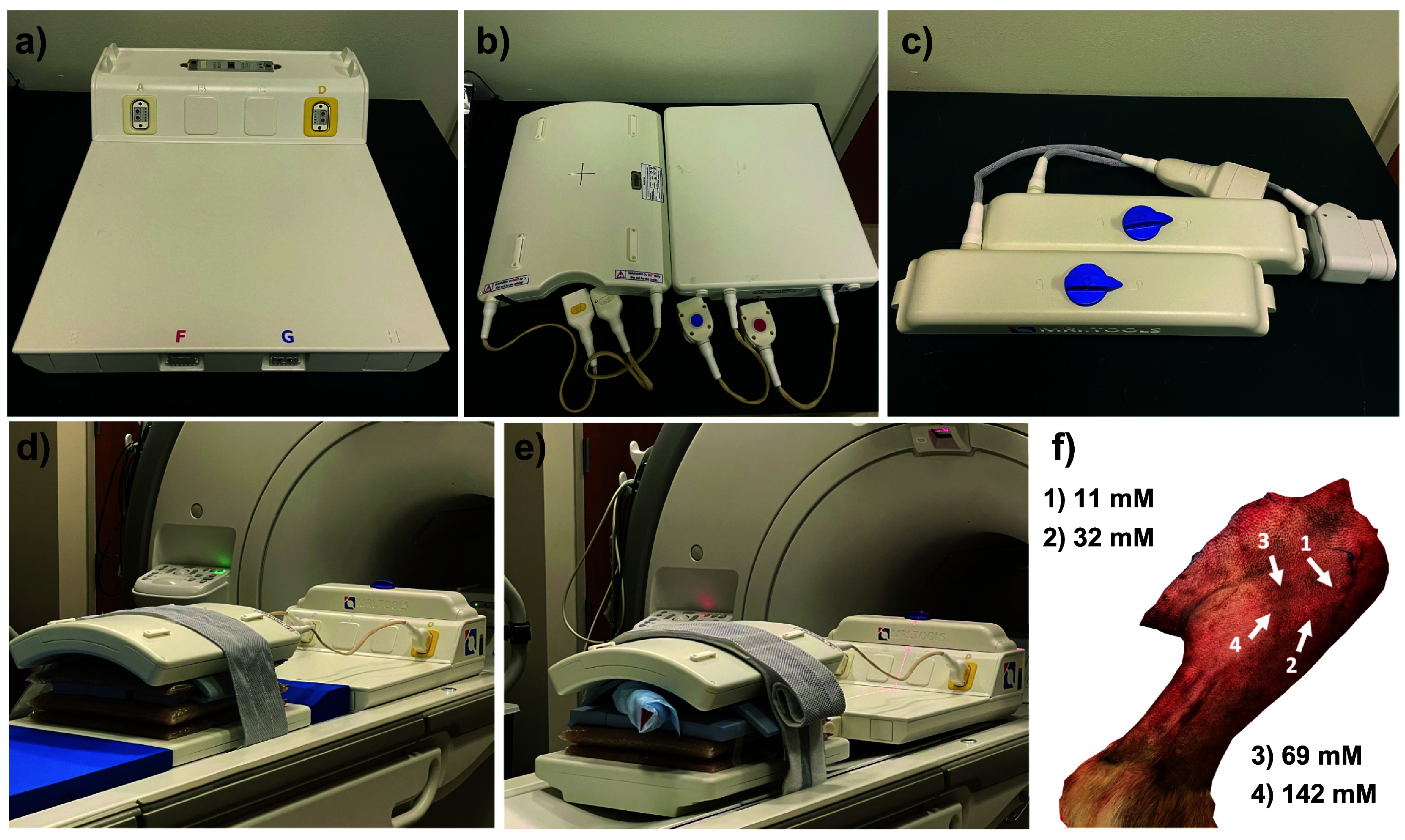
Dual-tuned ^1^H/^19^F MRI coil and setup. The 8-channel dual tuned ^1^H/^19^F transmit/receive (a) coil interface, (b) anterior and posterior coils, and (c) ^1^H and ^19^F modules. (d) The phantom measurements setup, (e) the canine *ex vivo* setup, and (f) the approximate location of the (n = 4) injection sites within the canine limb.

All measurements were conducted on a 3.0T Discovery MR750w MRI scanner (GE Healthcare, Waukesha, WI) with a quadrature 8-channel dual tuned ^1^H/^19^F torso coil (MRI Tools, Berlin, Germany) (figures 2(a)–(c)). The coil was designed that the two individual modules (^1^H/^19^F) can be interchanged to switch between ^1^H and ^19^F acquisition, without disturbing the setup. Agar phantom spacers (2% by weight, KCL: 30 mM) were made in 15 l plastic bags and were large enough to cover all the surfaces of the coil elements and ensure proper radio-frequency loading and spacing during phantom and *ex-vivo* canine limb measurements. Additionally, reference PFPE phantom vials (*N* = 4) and a highly concentrated vial (approx. 1.0 M) for calibration purposes (figure 2(d)) was placed inside the MRI setup. Reference PFPE vials were placed approximately 2–3 cm away from the surface of the anterior coil elements.

### 2.2. PFPE phantoms construction

PFPE (CS ATM 1000, Celsense; Pittsburgh, PA) phantoms were constructed by first mixing low melting point agarose powder (Sigma Aldrich; St Louis, MO) and water at 2% (w/w) and combining with PFPE at various concentrations. After dissolution of the agar, 1.5 ml aliquots of 16.0, 8.0, 4.0, 2.0 mg ml^−1^ of PFPE agar solution were added to 1.5 ml centrifuge tubes. Each vial was vortexed for 1 min to ensure adequate mixing and stored upright while cooling. The approximate ^19^F concentration of reference vials 1–4 are 166.1, 70.0, 41.5, and 18.3 mM, respectively. These concentration ranges were selected to represent a realistic concentration regime (micromolar), as addressed by Amiri *et al*, that is anticipated for *in vivo* cell tracking studies (Amiri *et al* 2015).

### 2.3. *In vitro* relaxation measurements

To characterize the *in vitro* longitudinal (*T*_1_) and transverse (*T*_2_) relaxation times for PFPE, a concentrated 2 ml vial of room temperature PFPE was placed inside the FOV along with the previously described MRI setup. To measure *T*_1_, a spectroscopic inversion recovery (IR) free induction decay (FID) sequence was prescribed with a TR = 3000 ms, 64 averages (NEX), and 12 inversion times ranging from 19 to 3000 ms. Similarly, the transverse relaxation time (*T*_2_) was measured using a spectroscopic spin-echo sequence with matching TR and NEX, and echo times ranging from 20 to 1000 ms. The *T*_1_ and *T*_2_ data were then processed by phasing, apodizing with a Lorentzian filter, zero-filling, and baseline correcting each spectrum. The main resonance peak of the processed PFPE spectra was then integrated (Mestrelab Mnova; Santiago, Spain) to give the total signal achieved. The total signal was then plotted against its respective IR or TE to generate the characteristic *T*_1_ and *T*_2_ relaxation curves. Using a nonlinear least-squares fitting, the *T*_1_ and *T*_2_ curves were fit to equations (1*a*) and (1*b*), respectively,

\begin{equation*}M\left( {{\mathrm{IR}}} \right) = {M_0}\left( {1 - A \cdot {{\mathrm{e}}^{ - \frac{{{\mathrm{IR}}}}{{{T_1}}}}}} \right)\end{equation*}

\begin{equation*}M\left( {{\mathrm{TE}}} \right) = {M_0} \cdot {{\mathrm{e}}^{ - \frac{{{\mathrm{TE}}}}{{{T_2}}}}} + C.\end{equation*}

### 2.4. Numerical simulations

To determine the parameters that would maximize signal acquisition efficiency for SPGR and bSSFP, numerical simulations were conducted via MATLAB 2020a (Mathworks, Natick, MA). The theoretical signal response was modeled for on-resonance acquisitions for a TR range of 1–100 ms and flip angles (FAs) spanning 1°–90° excitation with an instantaneous hard pulse at the previously measured room temperature *in vitro* relaxation times. The theoretical signal acquisition efficiency, $\varepsilon $, was calculated for the range of TR and FA, according to equation (2) (Flogel and Ahrens 2017):

\begin{equation*}\varepsilon \propto \frac{{\xi \left( {\alpha ,{\mathrm{TR}}} \right)}}{{\sqrt {{\mathrm{TR}}} }}\end{equation*}

where $\xi \left( {\alpha ,{\text{ TR}}} \right)$ is the maximum theoretical signal achieved by the sequence for a FA, $\alpha $, and repetition time, TR. The range of achievable TRs and subsequently, the minimum achievable TR, is dictated by the choice of BW at the given acquisition matrix. Heatmaps of the efficiency were generated to determine the theoretical Optimal FA and TR that maximized the efficiency and to compare the relative performance between the sequences.

### 2.5. Manual prescan (MPS) procedure

Before the acquisition of ^19^F images, a MPS procedure was performed to set the center frequency and the transmit gain (TG) setting necessary to achieve the desired FA. The standard shim calculated from the ^1^H acquisitions were applied for the ^19^F acquisitions. The central frequency was set using a 1D FID sequence. A nominal 90° selective RF pulse was used to excite a highly concentrated vial of PFPE. With the ^19^F power spectrum displayed, the operating frequency was then adjusted until the main PFPE peak was on resonance. Within the MPS of each sequence, a 1-cycle sinc pulse with a nominal FA of 90° and pulse width of 3.2 ms was used to excite the PFPE vial. Using the console-displayed power spectrum, the TG was increased until a maximum signal amplitude was achieved, signifying that a 90° flip has been achieved. After this 90° FA calibration, the ^19^F sequences can be prescribed, where the scan RF pulse will be automatically attenuated to the desired FA, according to equation (3),

\begin{equation*}\eta = - 200 \cdot {\log _{10}}\left[ {\frac{\alpha }{{{{90}^ \circ }{ }}}} \right]\end{equation*}

where $\eta $ is the required RF attenuation, in units of 0.1 dB, and $\alpha $ is our desired FA.

### 2.6. FA and receiver bandwidth validation

To ensure that the experimental optimal FAs are near their predicted locations, validation studies were performed. Conventional (^1^H) images were acquired using a 2D coronal SPGR with 12 slices where additional parameters can be found in table 1. Multinuclear-enabled 3D bSSFP, and SPGR sequences were prescribed with a matching excitation volume, 12 slice encoding steps, and minimum TR/TE. For each successive acquisition, the FA was increased to span FAs between 0°–30° and 30°–90° for SPGR and bSSFP, respectively. Additional scan parameters can be found in table 2. From the resulting images, the SNR within Vial 1 (166.1 mM), corrected for its multichannel bias, was calculated. The resulting FA with the greatest achieved SNR, for each sequence, was used in all future acquisitions at that combination of TR/TE and rBW.

**Table 1.** Relevant sequence parameters for the conventional (^1^H) acquisitions. Conventional/anatomic sequence parameters for both the phantom and *ex vivo* acquisitions.

Sequence	Application	Flip angle (°)	TR/TE (ms)	FOV (mm^2^)	Acq. matrix
2D SPGR	Phantom	20	34/3.288	400 × 400	256 × 256
2D FSE	*Ex vivo*	90	533/5.848		

**Table 2.** Relevant theoretically optimized flip angle for 3D SPGR and bSSFP-based sequences. Sequence parameters are listed for the theoretical sequence parameter validation.

Sequence	Predicted angle (°)	Flip angles tested (°)	TR/TE (ms)	rBW (kHz)
SPGR	11.8	5–30	9.2/3.9	10
bSSFP	63.9	40–90	8.4/4.1	

Similarly, various rBW values were then tested to determine which choice produced the most SNR-efficient acquisition, while minimizing any artifacts. For SPGR and bSSFP, receiver bandwidths ranging from 3 to 50 kHz were acquired with the minimum TR/TE at their respective predicted optimal FA. The qualitative image quality and both the SNR and SNR_eff_ were evaluated in Vial 1 to determine the most efficient and robust choice.

### 2.7. Pulse sequence comparisons

Sequences were compared for their maximum achieved SNR efficiency, using the theoretical imaging parameters calculated for SPGR and bSSFP. The sequences, along with a phase-cycled version of bSSFP (bSSFP-C), were prescribed with the previously determined optimal FA, TR/TE, and rBW; and NEX was set in order to achieve a clinically practical scan time of 10 min. Conventional 2D SPGR images were then acquired with 24 slices at 3 mm thickness (table 1). All ^19^F acquisitions were performed with an identical field-of-view, and resolution of 3.125 × 3.125 × 3mm^3^ (table 3). The resulting images were processed and the normalized SNR_eff_, hereafter ‘the sensitivity’, was assessed for each of the (*n* = 4) PFPE vials. Additionally, a linear regression was performed to extrapolate the empirical lower limit of detection for each of the sequences, where an SNR of 4 was defined as a conservative detection cutoff limit (Watts and Wang 2002).

**Table 3.** Relevant sequence parameters for 3D ^19^F SPGR, bSSFP, and bSSFP-C comparisons and *ex vivo* acquisitions. Sequence parameters are listed for both the optimized sequences for sensitivity assessment and comparison, and the *ex vivo* acquisitions.

Sequence	NEX	Flip angle (°)	TR/TE (ms)	rBW (kHz)	Acq. matrix	Resolution (mm^3^)	FOV (mm^3^)	Scan time
SPGR	13	12	9.2/3.9	10	$128 \times 128 \times 24$	$3.125 \times 3.125 \times 3$	$400 \times 400 \times 72$	9:42
bSSFP	15	65	8.4/4.1					10:14
bSSFP-C	7							9:32
bSSFP-C (*ex vivo*)	15				$128 \times 128 \times 20$		$400 \times 400 \times 60$	10:46

### 2.8. Image postprocessing

#### 2.8.1. Registration and vial segmentation

All image postprocessing and analysis was performed using MATLAB 2020a. Composite ^1^H/^19^F images were created using a rigid control point registration and masks for each of the vials in the FOV were segmented. From the resultant masks, the mean value, $\overline {{M_i}} $, was calculated for each vial within the FOV of the ^19^F images. Magnitude noise measurements were taken by placing a large rectangular ROI in a region devoid of signal within the original raw ^19^F volumes. The average noise value ($\bar N$) was then calculated. The noise mean was corrected for its chi-squared (${\chi ^2}$)-distribution, using the relationship $\bar N = 3.94\sigma $, in order to estimate the true noise variance (Constantinides *et al* 1997), $\sigma $. A lookup table was generated to correct the biased magnitude signal measurements for their non-central ${\chi ^2}$-distribution to arrive at an estimate of the true signal, $A$. The estimation of the true SNR as shown in equation (5),

\begin{equation*}{\mathrm{SNR}} = \frac{A}{\sigma },\end{equation*}

requires correction for biases in the noise signal as outlined further in the online supplement.

Given the nature of the biased ${\chi ^2}$ and non-central ${\chi ^2}$ distributed noise and signal, respectively, the overestimation of SNR increases at low SNR. For uncorrected SNR measurements of SNR < 6, the error can be as high as ∼25%; however, for SNR > 15 the error is less than 4% (Kellman and Mcveigh 2008). For this reason, correction factors will only be applied to measured SNRs $ \unicode{x2A7D} $ 15. Using the corrected true SNR estimate, the sensitivity was calculated for each detected vial according to equation (6):

\begin{equation*}S = \frac{{{\mathrm{SNR}}}}{{{C_{\mathrm{F}}} \cdot {V_{{\mathrm{vox}}}} \cdot \sqrt {{\mathrm{TR}} \cdot {\mathrm{NEX}}} }} = \frac{{{\mathrm{SN}}{{\mathrm{R}}_{{\mathrm{eff}}}}}}{{{C_{\mathrm{F}}} \cdot {V_{{\mathrm{vox}}}}}}{ }\left[ {{\mathrm{m}}{{\mathrm{s}}^{ - 0.5}}\mu {\mathrm{mo}}{{\mathrm{l}}^{ - 1}}} \right]\end{equation*}

where ${C_{\mathrm{F}}}$ is the concentration of PFPE in *μ*mol mm^−3^ for each vial, *V*_vox_ is the volume of the voxel, and NEX is the number of excitations or averages. A more detailed discussion of the registration, vial segmentation, and SNR corrections can be found in the online supplementary information and figure S1.

### 2.9. *Ex vivo* feasibility

An *ex vivo* canine limb was utilized in order to demonstrate the ability to detect PFPE within soft tissue. The limb was donated after a canine patient with osteosarcoma underwent tumor biopsy and limb amputation. Four 200 *μ*l PFPE injections of varying concentrations were injected into the rear canine limb along the proximal tibia. The concentrations of PFPE injected at sites 1–4 (figure 2(f)), were 11, 32, 69, and 142 mM in phosphate-buffered saline (PBS), respectively. Directly after injection, the limb was placed within the coil (figure 2(e)), with the agar bags and 2 reference vials, with concentrations of 41.5 and 18.3 mM taped to the surface of the limb.

Anatomic ^1^H images were acquired using a 2D coronal FSE with 4 echoes and 20 slices (table 1). Afterward, the ^1^H module was replaced with the ^19^F module and the multinuclear MPS was conducted without disturbing the imaging setup or limb. 3D ^19^F bSSFP-C images were acquired with matching imaging volume (table 3).

### 2.10. Statistics

All statistical analyses were performed using GraphPad Prism 9 (GraphPad Software; San Diego, CA). For comparison of three or more groups, a one-way ANOVA was performed. Any statistically significant findings underwent Tukey’s honestly significant differences post hoc tests. Any findings with a *p*-value < 0.05 were considered statistically significant. Reported measurements represent the mean value ± SD.

## 3. Results

### 3.1. Numerical simulations

**Figure 3.**
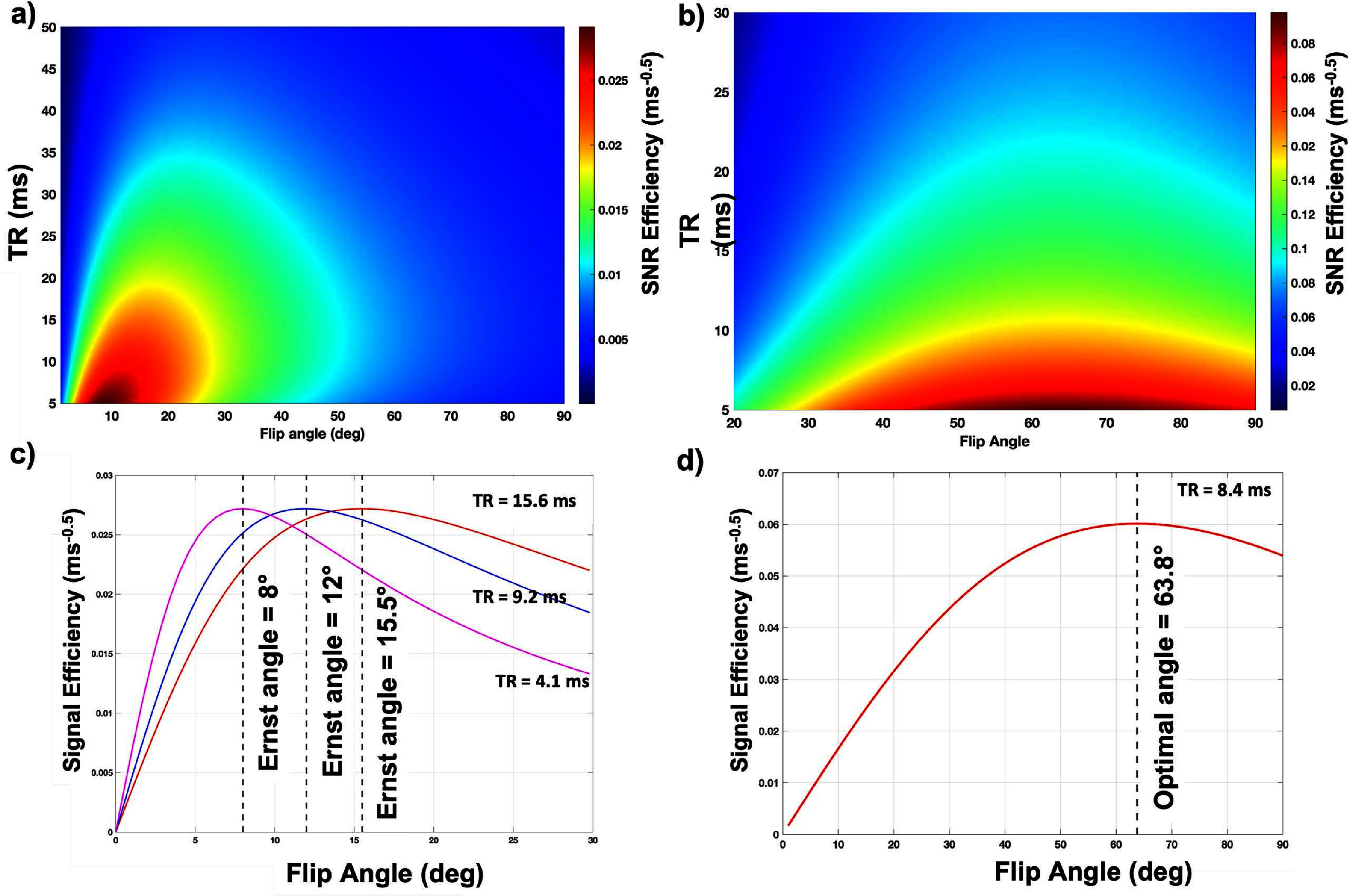
Bloch Simulations for SPGR and bSSFP of room temperature PFPE agent at 3T. A theoretical signal acquisition efficiency colormap was generated via Bloch simulations to determine the optimal theoretical flip angle and repetition time for (a) SPGR and (b) bSSFP pulse sequences for room temperature PFPE at 3T, where the scale bar represents the efficiency in units of ms^−0.5^. (c) Profiles of SNR efficiency at 3 different TRs were plotted against flip angle to demonstrate the dependency of TR on the theoretical optimal flip angle for SPGR sequences. (d) Profile of SNR efficiency for a TR to demonstrate the optimal flip angle for the bSSFP sequence. Maximal SNR efficiency is seen at minimal TR and between 8° and 16° for SPGR and 64° for bSSFP.

The numerical simulations describing the dependence of SNR_eff_ on TR and FA for SPGR and bSSFP are shown in figures 3(a) and (b). Simulations were conducted with PFPE relaxation times of *T*_1_/*T*_2_ = 424/165 ms, which were obtained from previously conducted *in vitro* measurements (online supplement figure S2). The results demonstrate that the signal acquisition efficiency for a fixed scan time will be maximized with the lowest possible TR. Empirically, TR_min_ is determined by the lowest possible rBW achievable without creating artifacts. Therefore, TR_min_ becomes a function of the chosen rBW for a set acquisition matrix. For bSSFP, a stable optimal angle of 63.8° that was generally independent of the choice of TR. SPGR’s optimal FA varied between 8*°* and 16° for TRs between 4.1 and 15.6 ms, the range of minimum available TRs on the 3T scanner with a fixed acquisition matrix of $128 \times 128 \times 30$.

### 3.2. FA validations

**Figure 4.**
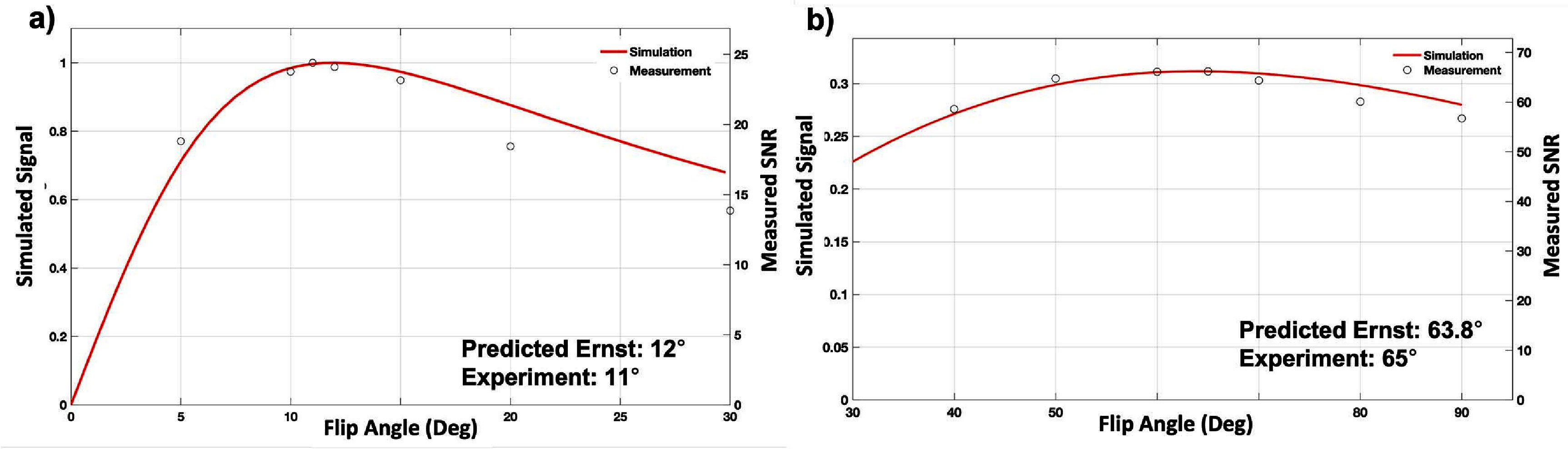
Flip angle validation for SPGR and bSSFP acquisitions of room temperature PFPE at 3T. Validation of theoretical optimal flip angle predictions for (a) SPGR with TR/TE = 9.2/3.9 ms and optimized flip angle for (b) bSSFP acquisitions (TR/TE = 8.4/4.1 ms), where SNR was measured in a single vial of room temperature PFPE agent across a range of flip angles. Theoretical predictions (red line left scale) and experimental results (black circles right scale) show close agreement with a predicted optimal flip angle of 12° and optimal flip angle of 63.8° and experimentally measured optimal flip angle of 11 and optimal flip angle of 65° for SPGR and bSSFP, respectively.

The experimental SNR measurements from the optimal FA and bSSFP optimized FA validation scans were then plotted against their manually calibrated FAs, along with the theoretical signal curve on a relative axis (figure 4). The results indicate experimental optimal FAs of 11° and 65° at a fixed TR/TE of 9.2/3.9 ms and 8.4/4.1 ms for SPGR and bSSFP, respectively. The results show close agreement with theoretical predictions.

**Figure 5.**
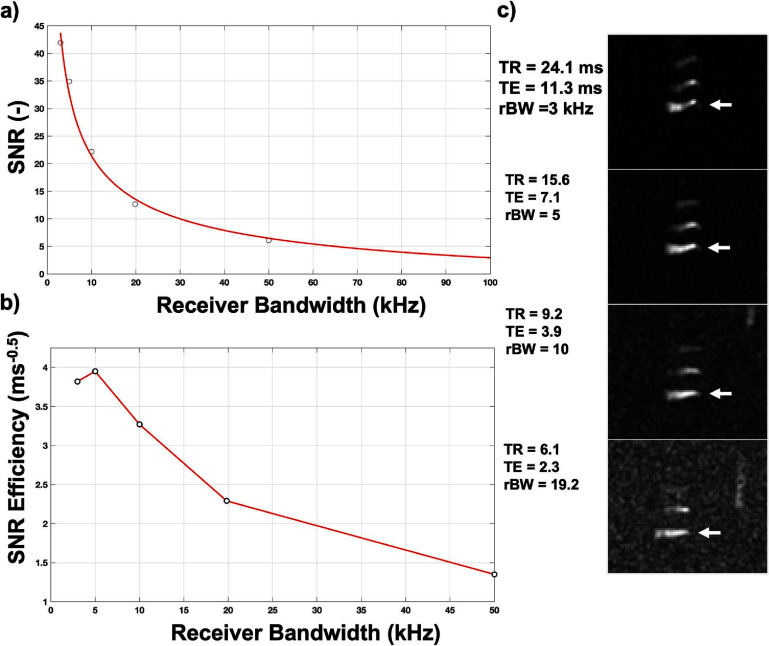
Selection of receiver bandwidth to maximize SNR efficiency for SPGR. (a) SNR and (b) SNR-efficiency for PFPE at 3T was measured at receiver bandwidths ranging from 3 to 50 kHz at the minimum TR/TE achievable. (c) ^19^F images demonstrate increased SNR with decreased rBW, but SNR-efficiency reaches a maximum at 5 kHz. Geometric distortions of the vials (white arrow) are also seen when rBW ⩽ 5 kHz; therefore, an rBW of 10 kHz was used to maximize SNR without introducing artifact.

These experimental FA values were used for the remaining experiments. The achieved SNR is increased with decreased rBW (figure 5(a)), as expected; however, the achieved SNR_eff_ (figure 5(b)) peaked at approximately 5 kHz. Although a rBW of 5 kHz had greater SNR_eff_ for SPGR, geometric distortions were visible, (figure 5(c)). bSSFP exhibited banding artifacts across rBW < 50 kHz; empirically, much more significant banding was seen at rBW < 10 kHz due to the increased dephasing because of the need for longer minimum TRs. Given the artifacts produced at low bandwidths, an rBW = 10 kHz presented the best tradeoff between SNR_eff_ and artifact and was used for the remainder of the studies.

### 3.3. Sequence comparisons

**Figure 6.**
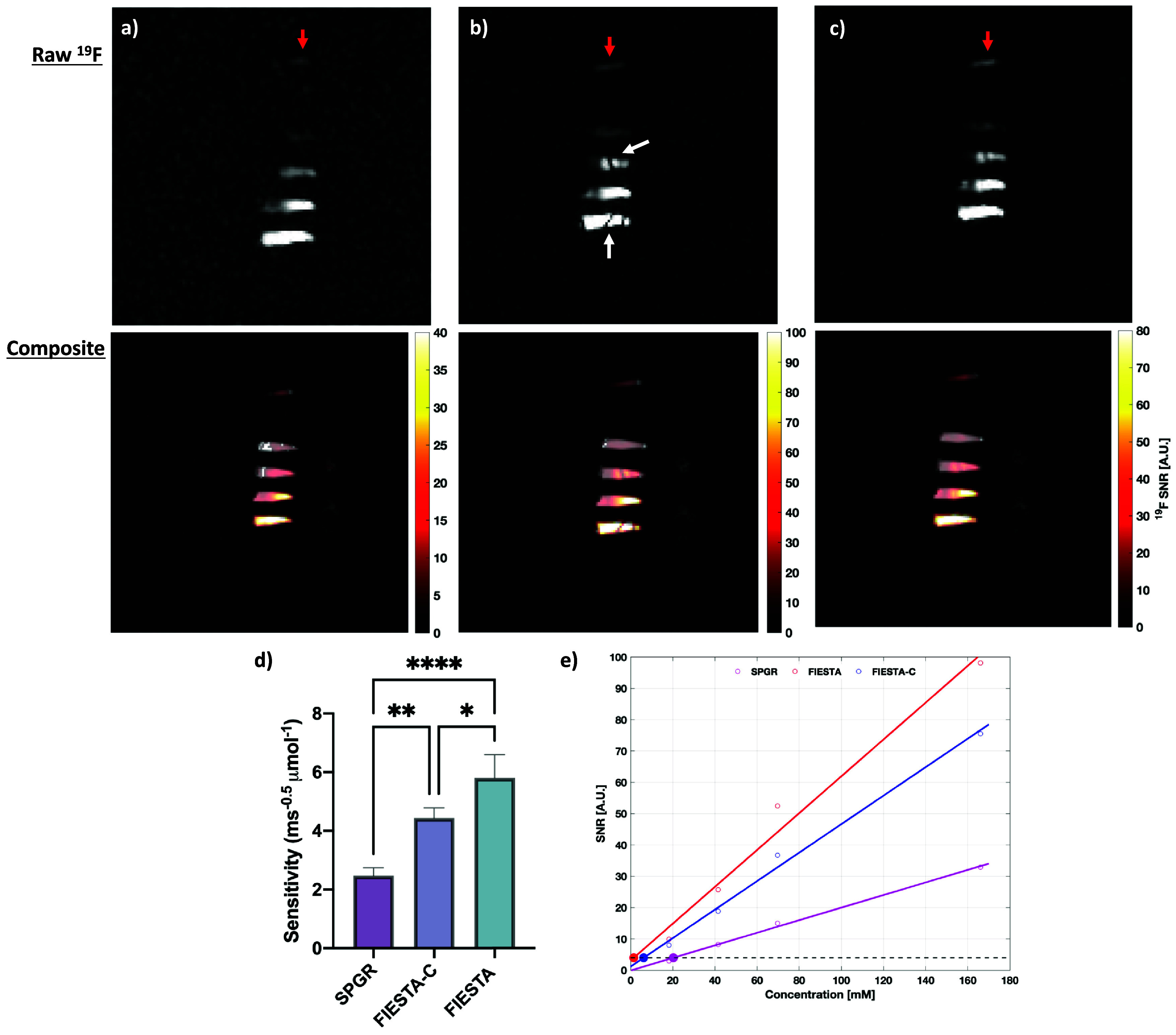
Assessment of SNR-efficiency SPGR, bSSFP, and bSSFP-C. Zoomed, registered coronal (a) SPGR, (b) bSSFP, and (c) bSSFP-C of raw ^19^F (top) and SNR-scaled composite ^1^H/^19^F images (bottom) were acquired using optimal parameters for a 10 min scan. (d) The SNR_eff_ was calculated for each of the 4 PFPE vials and compared using a non-parametric repeated measures ANOVA test. Tukey’s multiple comparisons test indicated a statistically significant increase in SNR_eff_ for bSSFP-C compared with an SPGR acquisition. The white arrow indicates a region of significant banding artifact and red arrows represent a small reference vial of PFPE. (e) Detection limits, where an SNR of 4 was considered the lower limit, were calculated for each sequence. Note: * represents p < 0.05, ** represents p < 0.01, and **** represents p < 0.0001.

The performance of SPGR, bSSFP, and bSSFP-C are compared in figures 6(a)–(c). SPGR produced images with minimal artifact but demonstrated lower SNR over all of the vials in the FOV. As expected from theory, bSSFP demonstrated very high levels of SNR across the vials but had visible off-resonance banding artifacts, whereas bSSFP-C produced images with reduced banding artifacts compared with its non-phase cycled counterpart but exhibited lower sensitivity. While bSSFP-C showed substantially improved SNR relative to SPGR, bSSFP produced the highest sensitivity of 5.81 ms^−0.5^ *μ*mol^−1^, followed by bSSFP-C with a mean sensitivity of 4.44 ms^−0.5^ *μ*mol^−1^, with SPGR having the lowest sensitivity of 2.47 ms^−0.5^ *μ*mol^−1^ (figure 6(d)). Results of the multiple comparisons determined that bSSFP-C produced significantly greater sensitivity than SPGR by a factor of 1.8 (*p* < 0.01) and that bSSFP outperformed both SPGR and bSSFP-C by a factor of 2.3 (*p* < 0.001) and 1.3 (*p* < 0.05), respectively. The lower limit of detection was then determined, as seen in figure 6(e), for each of the sequences. For a 10 min scan time under the optimized parameters, bSSFP, bSSFP-C, and SPGR, produced ^19^F detection limits of 1.5, 6.2, and 20.3 mM, respectively.

### 3.4. *Ex vivo* canine ^19^F bSSFP-C

**Figure 7.**
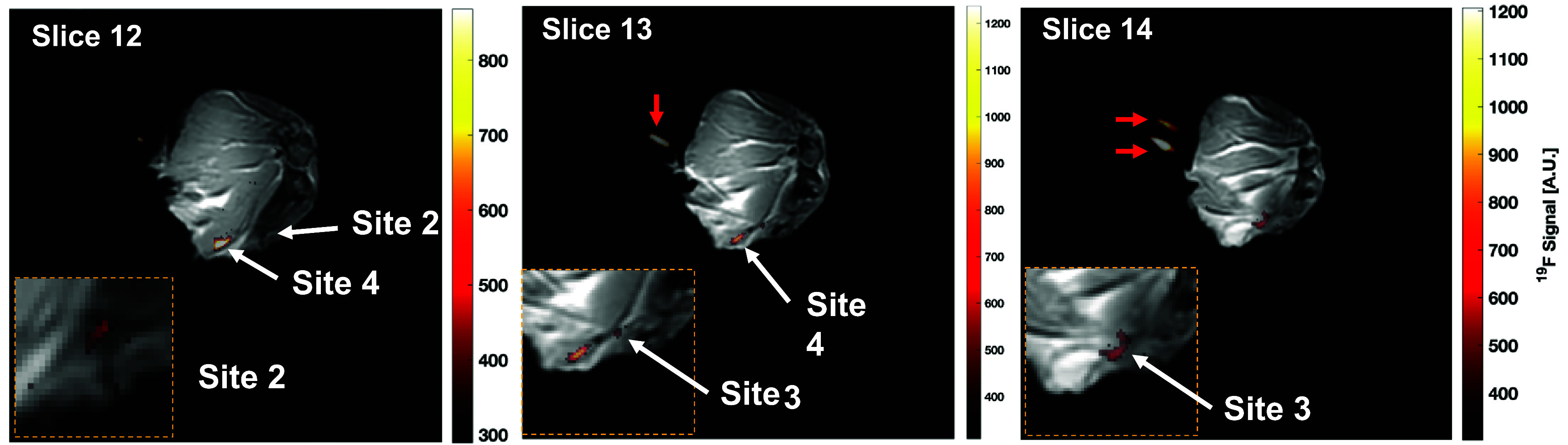
*Ex vivo* bSSFP-C composite images. ^1^H/^19^F bSSFP-C composite images of bSSFP-C acquisitions in an *ex vivo* canine limb. In slices 12–14, injections sites 2, 3, and 4 were detectible, as denoted with white arrows; however, site 1 was not visible in any of the acquisitions. Orange-dashed cutouts in each slice depict zoomed-in window leveled images of the injection sites. Red arrows depict the location of 2 reference vials taped to the limb. Each slice was window leveled individually for visualization purposes.

Results from the *ex vivo* canine ^19^F bSSFP-C MRI are shown in figure 7(a) and demonstrate the ability to detect injection sites 2–4, which ranged from 32 to 142 mM of PFPE in PBS. However, the 10 min acquisition was unable to detect signal from injection site 1 (11 mM) for any scan. A mean SNR of 4.8, 8.3, and 15.5 was achieved for injection sites 2 (32 mM), 3 (69 mM), and 4 (142 mM), where a linear regression (*R* ^2^ > 0.999) predicted a minimum detection limit of 24 mM of PFPE in this *ex vivo* imaging model.

## 4. Discussion

This study demonstrates a workflow to improve clinical translatability by optimizing the SNR-efficiency of product sequences for ^19^F MRI with a large field-of-view torso coil on a clinical 3T system. In this study, bSSFP sequences were shown to outperform SPGR in sensitivity for a common PFC nanoemulsion measured at the clinically relevant field strength of 3T. Specifically, experimentally verified relaxation times for PFPE, along with Bloch simulations were used to determine the optimal TR and FA to maximize the signal acquisition efficiency for PFPE using both SPGR and bSSFP sequences. Simulations were verified experimentally for feasible high performing choices of FA, TR, TE, and rBW, and compared head-to-head for a fixed 10 min scan time where relative sensitivity and detection limits were quantified and compared. The achieved sensitivities clearly show the benefit of a bSSFP approach for PFPE, and the use of the phase-cycled SSFP technique, or bSSFP-C to reduce banding artifact present across a wide range of rBW choices. bSSFP-C showed a modest reduction in sensitivity compared to bSSFP but demonstrated an approximately 1.8-fold increase in sensitivity compared to SPGR and the ability to reduce the banding artifacts.

PFPE was chosen for this study due to its high ^19^F content, the lowest *T*_1_ of the more common PFC agents, and exhibits a relatively simple resonance structure. For this reason and its relatively rapid clearance, the PFPE agent has been utilized in various cell tracking and inflammation studies (Balducci *et al* 2012, Weibel *et al* 2013, Vasudeva *et al* 2014). However, an alternative agent, PFCE, has a single resonance line and high ^19^F content and has been used in many preclinical studies. Moreover, Colotti *et al* showed that PFCE has a greater *T*_2_/*T*_1_ relaxation ratio, which makes it more suitable for a bSSFP approach (Colotti *et al* 2017). Potential toxicity due to PFCE’s long persistence in the liver and spleen precludes its use in clinical imaging studies (Jacoby *et al* 2014) although recent work has shown the ability to reduce the *in vivo* biological half-life 15-fold by modifying the nanoemulsion structure making this agent of potential interest for future clinical development (Staal *et al* 2020). Taken together, these findings show that more work is required to identify proper PFC agent which will optimize cell labeling while limiting patient toxicity. Nevertheless, this work demonstrates that ^19^F MRI is feasible with clinical multi-channel body coils and standard pulse sequences.

FA calibration utilizing a modified version of vendor’s built-in MPS, was reliably achieved for the commercially available PFPE agent and multi-channel ^19^F torso coil which was able to realize stable optimized FA’s matched to predicted values within 1°–1.5°. Practical tradeoffs in rBW and TR in the presence of B_0_ inhomogeneities were demonstrated, with banding artifact reduced effectively using the bSSFP-C acquisition. While the acquisition of multiple phase cycles improved image quality for bSSFP-C, there is a consequent increase in minimum acquisition time and reduced sensitivity due to the MIP used to combine the two phase-cycles. An alternate approach to boost the achieved sensitivity and image quality would seek to combine 2 or more phase cycles using a sum of squares approach (Elliott *et al* 2007, Mallett and Foster 2011).

A reduction in sensitivity was seen moving to an *ex vivo* model where PFPE was injected into an amputated canine limb. However, as the *ex vivo* PFPE detection limit is within a biologically relevant range, these results support the motivation to advance this work with ACT using an *in vivo* canine model. While translating ^19^F MRI for tracking of ACT to a clinical setting has its technical challenges demanding careful design and optimization to improve sensitivity.

This presented work also has its limitations. First, the theoretical modeling and validations of sequence parameters are demonstrated for PFPE at room temperature but given the relaxation times’ temperature and tissue (*in vitro* versus *in vivo*) dependence, the optimal parameters will likely shift accordingly. However, the provided methodology could be extended to a more relevant biological temperature, and different PFC emulsions, such as PFCE and even different X-nuclei as the basic optimization workflow will be similar. Second, although the flip calibration using the MPS was performed in as little as 5–10 min, fully automated FA calibration would be helpful for clinical research studies (Goette *et al* 2015b). Lastly, B_1_ inhomogeneities, which could lead to variable signal response in the FOV, were not taken into consideration in this study. However, given the linearity of measured sensitivity across the four spatially separated ^19^F samples, this affect is assumed to be small within the quadrature B1-field of the phased array coil. Nevertheless, the ^1^H signal of a ^1^H/^19^F dual tuned coil or an automated Bloch–Siegert approach (Schulte *et al* 2011) could be used in the future in order to conduct regional B_1_-mapping (Goette *et al* 2015b).

In conclusion, this work demonstrates a workflow for assessing performance of ^19^F MRI sequences. A greater relative SNR-efficiency of bSSFP was found compared to bSSFP-C and SPGR sequences for PFPE acquisitions at 3T. Additionally, the bSSFP-C sequence, was able to detect a PFC nanoemulsion in an *ex vivo* canine limb and a large FOV torso coil with minimal banding artifacts within clinically acceptable scan times at 3T. Taken together, this work demonstrates the feasibility of clinical whole-body ^19^F MRI which is necessary for *in-vivo* cell tracking investigations and provides the foundation for future studies to demonstrate the ability to detect PFC-labeled adoptively transferred NK cells in an *in vivo* canine osteosarcoma model.

## Data Availability

The data cannot be made publicly available upon publication because they are not available in a format that is sufficiently accessible or reusable by other researchers. The data that support the findings of this study are available upon reasonable request from the authors.

## References

[pmbad4d50bib1] Ahrens E T, Helfer B M, O’Hanlon C F, Schirda C (2014). Clinical cell therapy imaging using a perfluorocarbon tracer and fluorine-19 MRI. Magn. Reson. Med..

[pmbad4d50bib2] Amiri H, Srinivas M, Veltien A, van Uden M J, de Vries I J M, Heerschap A (2015). Cell tracking using 19F magnetic resonance imaging: technical aspects and challenges towards clinical applications. Eur. Radiol..

[pmbad4d50bib3] Anon (2013). Preclinical assessment of investigational cellular and gene therapy products | FDA. https://www.fda.gov/regulatory-information/search-fda-guidance-documents/preclinical-assessment-investigational-cellular-and-gene-therapy-products.

[pmbad4d50bib4] Balducci A, Helfer B M, Ahrens E T, O’Hanlon C F, Wesa A K (2012). Visualizing arthritic inflammation and therapeutic response by fluorine-19 magnetic resonance imaging (19F MRI). J. Inflamm..

[pmbad4d50bib5] Bernstein M, King K, Zhou X (2005). Handbook of MRI Pulse Sequences.

[pmbad4d50bib6] Bönner F (2015). Monocyte imaging after myocardial infarction with 19FMRI at 3 T: a pilot study in explanted porcine hearts. Eur. Heart J. Cardiovascular Imaging.

[pmbad4d50bib7] Bouchlaka M N, Ludwig K D, Gordon J W, Kutz M P, Bednarz B P, Fain S B, Capitini C M (2016). 19F-MRI for monitoring human NK cells in vivo. Oncoimmunology.

[pmbad4d50bib8] Chapelin F, Gao S, Okada H, Weber T G, Messer K, Ahrens E T (2017). Fluorine-19 nuclear magnetic resonance of chimeric antigen receptor T cell biodistribution in murine cancer model. Sci. Rep..

[pmbad4d50bib9] Chavhan G B, Babyn P S, Jankharia B G, Cheng H L M, Shroff M M (2008). Steady-state MR imaging sequences: physics, classification, and clinical applications. Radiographics.

[pmbad4d50bib10] Colotti R, Bastiaansen J A M, Wilson A, Flögel U, Gonzales C, Schwitter J, Stuber M, van Heeswijk R B (2017). Characterization of perfluorocarbon relaxation times and their influence on the optimization of fluorine-19 MRI at 3 tesla. Magn. Reson. Med..

[pmbad4d50bib11] Constantinides C D, Atalar E, McVeigh E R (1997). Signal-to-noise measurements in magnitude images from NMR phased arrays. Magn. Reson. Med..

[pmbad4d50bib12] Darçot E, Colotti R, Pellegrin M, Wilson A, Siegert S, Bouzourene K, Yerly J, Mazzolai L, Stuber M, van Heeswijk R B (2019). Towards quantification of inflammation in atherosclerotic plaque in the clinic—characterization and optimization of fluorine-19 MRI in mice at 3 T. Sci. Rep..

[pmbad4d50bib13] Dubois V P, Sehl O C, Foster P J, Ronald J A (2022). Visualizing CAR-T cell immunotherapy using 3 tesla fluorine-19 MRI. Mol. Imaging Biol..

[pmbad4d50bib14] Elliott A M, Bernstein M A, Ward H A, Lane J, Witte R J (2007). Nonlinear averaging reconstruction method for phase-cycle SSFP. Magn. Reson. Imaging.

[pmbad4d50bib15] Flogel U, Ahrens E (2017). Fluorine Magnetic Resonance Imaging.

[pmbad4d50bib16] Goette M J, Keupp J, Rahmer J, Lanza G M, Wickline S A, Caruthers S D (2015a). Balanced UTE-SSFP for 19F MR imaging of complex spectra. Magn. Reson. Med..

[pmbad4d50bib17] Goette M J, Lanza G M, Caruthers S D, Wickline S A (2015b). Improved quantitative 19F MR molecular imaging with flip angle calibration and B1-mapping compensation. J. Magn. Reson. Imaging.

[pmbad4d50bib18] Hargreaves B A, Vasanawala S S, Pauly J M, Nishimura D G (2001). Characterization and reduction of the transient response in steady-state MR imaging. Magn. Reson. Med..

[pmbad4d50bib19] Hargreaves B (2012). Rapid gradient-echo imaging. J. Magn. Reson. Imaging.

[pmbad4d50bib20] Hingorani D V, Chapelin F, Stares E, Adams S R, Okada H, Ahrens E T (2020). Cell penetrating peptide functionalized perfluorocarbon nanoemulsions for targeted cell labeling and enhanced fluorine-19 MRI detection. Magn. Reson. Med..

[pmbad4d50bib21] Jacoby C, Temme S, Mayenfels F, Benoit N, Krafft M P, Schubert R, Schrader J, Flögel U (2014). Probing different perfluorocarbons for in vivo inflammation imaging by 19F MRI: image reconstruction, biological half-lives and sensitivity. NMR Biomed..

[pmbad4d50bib22] Janjic J M, Ahrens E T (2009). Fluorine-containing nanoemulsions for MRI cell tracking. Nanomedicine and Nanotechnol..

[pmbad4d50bib23] Kellman P, Mcveigh E R (2008). SNR unit recon. Magn. Reson. Med..

[pmbad4d50bib24] Lechuga L M, Ludwig K D, Forsberg M H, Walker K L, Capitini C M, Fain S B (2021). Detection and viability of murine NK cells in vivo in a lymphoma model using fluorine-19 MRI. NMR Biomed..

[pmbad4d50bib25] Makela A V, Foster P J (2018). Imaging macrophage distribution and density in mammary tumors and lung metastases using fluorine-19 MRI cell tracking. Magn. Reson. Med..

[pmbad4d50bib26] Mallett C L, Foster P J (2011). Optimization of the balanced steady state free precession (bSSFP) pulse sequence for magnetic resonance imaging of the mouse prostate at 3T. PLoS One.

[pmbad4d50bib27] Melero I, Rouzaut A, Motz G T, Coukos G (2014). T-cell and NK-cell infiltration into solid tumors: a key limiting factor for efficacious cancer immunotherapy. Cancer Discovery.

[pmbad4d50bib28] Newick K, O’Brien S, Moon E, Albelda S M (2017). CAR T cell therapy for solid tumors. Annu. Rev. Med..

[pmbad4d50bib29] Rosenberg S A (2001). Progress in human tumour immunology and immunotherapy. Nature.

[pmbad4d50bib30] Rosenberg S A, Restifo N P, Yang J C, Morgan R A, Dudley M E (2008). Adoptive cell transfer: a clinical path to effective cancer immunotherapy. Nat. Rev. Cancer.

[pmbad4d50bib31] Rothe M, Jahn A, Weiss K, Hwang J H, Szendroedi J, Kelm M, Schrader J, Roden M, Flögel U, Bönner F (2019). In vivo 19 F MR inflammation imaging after myocardial infarction in a large animal model at 3 T. Magn. Reson. Mater. Phys. Biol. Med..

[pmbad4d50bib32] Schulte R F, Sacolick L, Deppe M H, Janich M A, Schwaiger M, Wild J M, Wiesinger F (2011). Transmit gain calibration for nonproton MR using the Bloch-Siegert shift. NMR Biomed..

[pmbad4d50bib33] Seymour L (2017). iRECIST: guidelines for response criteria for use in trials testing immunotherapeutics. Lancet Oncol..

[pmbad4d50bib34] Somanchi S S, Kennis B A, Gopalakrishnan V, Lee D A, Bankson J A (2016). In vivo 19F-magnetic resonance imaging of adoptively transferred NK cells. Natural Killer Cells: Methods and Protocols.

[pmbad4d50bib35] Srinivas M, Morel P A, Ernst L A, Laidlaw D H, Ahrens E T (2007). Fluorine-19 MRI for visualization and quantification of cell migration in a diabetes model. Magn. Reson. Med..

[pmbad4d50bib36] Srinivas M, Turner M S, Janjic J M, Morel P A, Laidlaw D H, Ahrens E T (2009). In vivo cytometry of antigen-specific T cells using 19F MRI. Magn. Reson. Med..

[pmbad4d50bib37] Sta Maria N S, Barnes S R, Jacobs R E (2014). In vivo monitoring of natural killer cell trafficking during tumor immunotherapy. Magn. Reson. Insights.

[pmbad4d50bib38] Staal A H J (2020). In vivo clearance of 19F MRI imaging nanocarriers is strongly influenced by nanoparticle ultrastructure. Biomaterials.

[pmbad4d50bib39] Temme S, Bönner F, Schrader J, Flögel U (2012). 19F magnetic resonance imaging of endogenous macrophages in inflammation. Wiley Interdiscip. Rev. Nanomed. Nanobiotechnol..

[pmbad4d50bib40] Turtle C J (2016). CD19 CAR-T cells of defined CD4+:CD8+ composition in adult B cell ALL patients. J. Clin. Invest..

[pmbad4d50bib41] Varani M, Auletta S, Signore A, Galli F (2019). State of the art of natural killer cell imaging: a systematic review. Cancers.

[pmbad4d50bib42] Vasudeva K, Andersen K, Zeyzus-Johns B, Hitchens T K, Patel S K, Balducci A, Janjic J M, Pollock J A (2014). Imaging neuroinflammation in vivo in a neuropathic pain rat model with near-infrared fluorescence and 19 magnetic resonance. PLoS One.

[pmbad4d50bib43] Waiczies H (2011). Perfluorocarbon particle size influences magnetic resonance signal and immunological properties of dendritic cells. PLoS One.

[pmbad4d50bib44] Watts R, Wang Y (2002). k-space interpretation of the rose model: noise limitation on the detectable resolution in MRI. Magn. Reson. Med..

[pmbad4d50bib45] Weibel S (2013). Imaging of intratumoral inflammation during oncolytic virotherapy of tumors by 19F-magnetic resonance imaging (MRI). PLoS One.

[pmbad4d50bib46] Zhong J, Mills P H, Hitchens T K, Ahrens E T (2013). Accelerated fluorine-19 MRI cell tracking using compressed sensing. Magn. Reson. Med..

